# The Use of an Omental Flap in the Management of Complex Thoracic Aortic Infections

**DOI:** 10.7759/cureus.111366

**Published:** 2026-06-23

**Authors:** Zachary Brennan, Tyler J Wallen, Thomas M Beaver, Eric I Jeng, Tomas D Martin, George J Arnaoutakis

**Affiliations:** 1 Department of Cardiac Surgery, Smidt Heart Institute, Cedars-Sinai Medical Center, Los Angeles, USA; 2 Department of Surgery, Division of Cardiovascular Surgery, Penn Medicine, Philadelphia, USA; 3 Department of Surgery, Division of Cardiovascular Surgery, University of Florida College of Medicine, Gainesville, USA; 4 Department of Surgery and Perioperative Care, Division of Cardiovascular and Thoracic Surgery, Dell Medical School, The University of Texas at Austin, Austin, USA

**Keywords:** aortic surgery, omental flap, prosthetic infection, recurrent endocarditis, thoracic aortic graft infection

## Abstract

Purpose

Recurrent valvular endocarditis and thoracic aortic graft infection (TAGI) present complex clinical problems with difficult management options and poor reported outcomes. Omental flap coverage has been described for these infections, but published experience remains limited. The purpose of this study was to describe our institutional experience with staged mediastinal antibiotic irrigation followed by delayed mediastinal omental flap coverage.

Methods

We performed a retrospective review of a prospectively maintained institutional database. Adult patients treated at a single institution from January 1, 2017, through December 31, 2019, were included if they underwent operative management for recurrent valvular endocarditis and/or TAGI with mediastinal antibiotic irrigation followed by planned, delayed mediastinal omental flap coverage. Patients were excluded if they were managed nonoperatively, underwent surgery without this staged strategy, had isolated superficial sternal wound infection without graft or valvular involvement, or had insufficient documentation to determine 30-day outcomes.

Results

Seventeen patients were treated with mediastinal antibiotic irrigation followed by delayed omental flap coverage. Thirty-day mortality was 1/17 (5.9%). Major complications within 30 days occurred in 4/17 (23.5%), including renal failure requiring hemodialysis in 2/17 (11.8%), stroke in 1/17 (5.9%), and reoperation for recurrent infection in 1/17 (5.9%). The composite 30-day major adverse event rate, defined as death, stroke, renal failure requiring hemodialysis, or reoperation for recurrent infection, was 5/17 (29.4%).

Conclusions

In this single-center series, staged mediastinal antibiotic irrigation followed by delayed omental flap coverage was feasible and was associated with acceptable short-term mortality and complication rates. Further study is needed to determine the optimal timing and patient selection criteria for omental flap coverage in this setting.

## Introduction

Recurrent valvular endocarditis and thoracic aortic graft infection (TAGI) present complex and difficult clinical situations in patients who have previously undergone aortic repair [1,2]. Graft infection is rare, with a reported incidence of approximately 1% to 3%, but mortality varies widely and has been reported between 25% and 88% [3,4]. While these complications are uncommon, management can be difficult, and multiple methods of repair have been described with varying degrees of success [5,6]. The current literature remains insufficient to establish a uniform standard of treatment for this complication, which carries significant morbidity and mortality [7].

The use of an omental flap to treat endocarditis and/or TAGI has been described previously. A paper as early as 1987 described the use of an omental flap in two patients to minimize the risk of infection and protect the vascular prosthesis after mediastinal infection [8]. Since that time, several single-center series, multicenter reports, and case-based studies have described omental flap coverage, graft replacement, graft preservation, staged reconstruction, and adjunctive antimicrobial strategies in this high-risk population [9-20].

In light of the difficult nature of these conditions and their associated outcomes, we sought to examine our institutional outcomes using mediastinal antibiotic irrigation followed by delayed omental flap coverage for patients with recurrent endocarditis and/or TAGI.

## Materials and methods

Study design and setting

The Institutional Review Board of the University of Florida approved this study (IRB202000163). We performed a retrospective review of a prospectively maintained institutional database at a single center. The study period included January 1, 2017, through December 31, 2019.

Patient selection

We included all adult patients (18 years or older) who underwent operative management for recurrent valvular endocarditis and/or TAGI in whom the institutional treatment strategy included mediastinal antibiotic irrigation followed by planned, delayed mediastinal omental flap coverage. The index operation was defined as the operation performed to treat the infected graft, recurrent endocarditis, prosthetic valve infection, or combined pathology before delayed omental flap coverage.

Exclusion criteria

Patients were excluded if they (1) were managed nonoperatively, (2) underwent operative management without mediastinal antibiotic irrigation and delayed omental flap coverage, (3) had isolated superficial sternal wound infection without concern for graft or valvular involvement, or (4) had insufficient documentation to determine 30-day outcomes.

Data collection and variables

Data were abstracted from the institutional database and supplemented by review of operative reports, inpatient records, discharge documentation, and follow-up notes when needed. Collected variables included demographics (age, sex, and race/ethnicity), comorbidities, operative characteristics (cardiopulmonary bypass time and aortic cross-clamp time), transfusion requirements, postoperative ventilator time, hospital length of stay, and postoperative outcomes. Primary outcomes were 30-day mortality and major postoperative complications within 30 days, including stroke, renal failure requiring hemodialysis, and reoperation for recurrent infection. Secondary outcomes included blood product utilization, duration of postoperative mechanical ventilation, hospital length of stay, follow-up mortality after discharge, cardiovascular mortality during follow-up, and intra-abdominal complications related to omental flap harvest.

Definitions and follow-up

Stroke was defined as a new postoperative neurologic deficit diagnosed clinically and/or radiographically. Renal failure was defined as postoperative renal failure requiring hemodialysis. Reoperation for recurrent infection was defined as any subsequent operation within 30 days performed for persistent or recurrent mediastinal, valvular, or graft-related infection. Follow-up time was calculated from the index operation date to the most recent clinical follow-up or death.

Statistical analysis

Given the descriptive intent and small cohort size, statistical analysis was limited to descriptive statistics. Continuous variables are reported as mean ± standard deviation and, when appropriate, median values. Categorical variables are reported as counts and percentages. No comparative hypothesis testing was performed because there was no control group, and the cohort size was limited.

Operative technique

The index operation was performed according to the presenting pathology. A summary of index operations is provided in the Results section. After completion of the index operation, an 18 Fr red rubber catheter (Becton Dickinson; Franklin Lakes, New Jersey, USA) was introduced into the mediastinum through a small incision on the right chest. The catheter was used for antibiotic irrigation beginning on postoperative day 1 after hemostasis was confirmed. The most frequently used agent was gentamicin (500 mg in 1 L), infused continuously over 24 hours.

**Figure 1:**
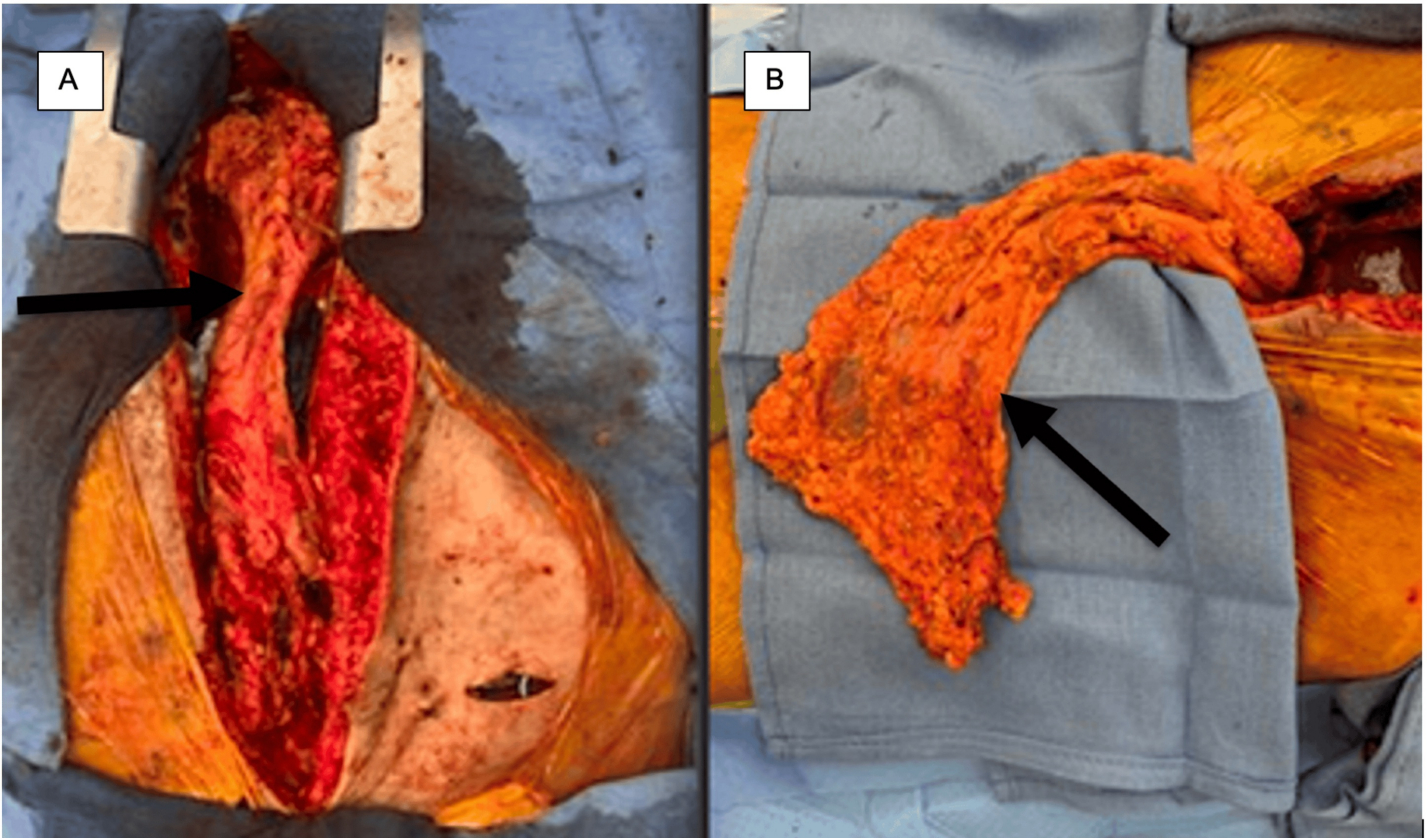
Omental Flap Harvest (left panel, A) and Mediastinal Transposition (right panel, B)

Patients then returned to the operating room for mediastinal exploration and washout when hemodynamically stable. The lower portion of the incision was carried down to the mid-abdomen, and the abdomen was explored. A flap of omentum was harvested while maintaining the vascular pedicle. Representative intraoperative images are shown in Figure 1A and Figure 1B. The omental flap was then transposed into the mediastinum and sutured into place at the superior aspect of the operative field. The abdomen and chest were then closed in standard fashion.

## Results

Seventeen patients met the study inclusion criteria. Presenting indications for surgery are listed in Table 1, and index operations performed before delayed omental flap coverage are listed in Table 2. Five patients presented for graft replacement, five patients presented for recurrent endocarditis, three patients presented for graft replacement and recurrent endocarditis, and four patients presented for prosthetic valve infection requiring replacement. The index operations included graft replacement, redo ascending aorta repair, redo aortic root procedures, redo aortic valve replacement, redo aortic root with graft replacement, redo ascending aorta with aortic root and graft replacement, and replacement of infected prosthetic valves.

**Table 1:** Indications for Surgery

Indication	n	%
Graft replacement	5	29.4
Recurrent endocarditis	5	29.4
Graft replacement and recurrent endocarditis	3	17.6
Prosthetic valve infection	4	23.5

**Table 2:** Index Operation

Index operation	n	%
Graft replacement	1	5.9
Redo ascending aorta	3	17.6
Redo aortic root	2	11.8
Redo aortic valve replacement	1	5.9
Redo aortic valve replacement and root	2	11.8
Redo aortic root and graft replacement	3	17.6
Redo ascending aorta, aortic root, and graft replacement	1	5.9
Replace infected prosthetic valve	4	23.5

Demographics and baseline characteristics are described in Table 3. The average age was 60 ± 13.9 years. All 17 patients were male. Thirteen patients were Caucasian, one was African American, one was Hispanic/Latino, one patient had race listed as other, and one patient declined to identify race. Comorbidities included diabetes in 4 patients, hypertension in 14 patients, dyslipidemia in 14 patients, liver disease in 5 patients, former smoking in 8 patients, current smoking in 4 patients, previous cerebrovascular accident in 3 patients, chronic obstructive pulmonary disease (COPD) in 7 patients, and previous cardiac surgery in all 17 patients.

**Table 3:** Baseline Demographics and Comorbidities COPD: chronic obstructive pulmonary disease

Variable	Value
Age (years)	60 ± 13.9
Male	17 (100%)
Caucasian	13 (76.5%)
African American	1 (5.9%)
Hispanic/Latino	1 (5.9%)
Other race	1 (5.9%)
Patient declined to identify race	1 (5.9%)
Diabetes	4 (23.5%)
Hypertension	14 (82.4%)
Dyslipidemia	14 (82.4%)
Liver disease	5 (29.4%)
Former smoker	8 (47.1%)
Current smoker	4 (23.5%)
Previous cerebrovascular accident	3 (17.6%)
COPD	7 (41.2%)
Previous cardiac surgery	17 (100%)

Operative data and outcomes are described in Table 4. The average cardiopulmonary bypass (CPB) time was 253.8 ± 114.1 minutes, and the average aortic cross-clamp time was 185.8 ± 86.3 minutes. The average follow-up time was 11.35 ± 11.09 months after the index operation.

**Table 4:** Operative Data and Outcomes CPB: cardiopulmonary bypass; FFP: fresh frozen plasma; RBC: red blood cell

Variable	Value
Average CPB (min)	253.8 ± 114.1
Average cross clamp (min)	185.8 ± 86.3
30-day mortality	1 (5.9%)
Renal failure with hemodialysis	2 (11.8%)
Reoperation	1 (5.9%)
Reoperation for recurrent infection	1 (5.9%)
Stroke	1 (5.9%)
Patients requiring blood products	17 (100%)
Average RBC units (17 patients)	5.6 ± 3.2
Average FFP units (7 patients)	2.8 ± 2.1
Average platelet units (17 patients)	2.2 ± 0.72
Average cryoprecipitate units (14 patients)	1.9 ± 0.77
Multi-system organ failure	0 (0%)
Required intubation postoperatively	8 (47.1%)
Postoperative ventilator time (min)	552.87 ± 314.27
Days from admission to discharge	38.9 ± 26.0
Time to follow-up (months)	11.35 ± 11.09
Follow-up mortality (among hospital survivors, n=16)	2 (12.5%)
Cardiovascular mortality at follow-up (among hospital survivors, n=16)	0 (0%)

The cohort had a 30-day mortality rate of 1/17 (5.9%). Major complications within 30 days occurred in 4/17 (23.5%) patients, including renal failure requiring hemodialysis in 2/17 (11.8%), stroke in 1/17 (5.9%), and reoperation for recurrent infection in 1/17 (5.9%). The composite rate of major adverse events within 30 days, defined as death, stroke, renal failure requiring hemodialysis, or reoperation for recurrent infection, was 5/17 (29.4%).

All 17 patients required blood products. Seventeen patients required red blood cell (RBC) units, with an average of 5.6 ± 3.2 units and a median of five units. Seven patients required fresh frozen plasma (FFP), with an average of 2.8 ± 2.1 units and a median of two units. Seventeen patients required platelets, with an average of 2.2 ± 0.72 units and a median of two units. Fourteen patients required cryoprecipitate, with an average of 1.9 ± 0.77 units and a median of two units.

Eight patients (47.1%) were intubated when they left the operating room, with an average postoperative ventilator time of 552.87 ± 314.27 minutes. The average time from admission to discharge was 38.9 ± 26.0 days. Two patients died during the follow-up period after discharge, neither from cardiovascular causes. There were no intra-abdominal complications related to omental flap harvest.

## Discussion

TAGIs, recurrent endocarditis, and prosthetic valve infections present challenging clinical problems with high associated morbidity and mortality. Various treatment modalities have been attempted to manage this complex problem, but complication rates remain high. One method that has been utilized is omental flap coverage after the index operation is performed to address the presenting pathology.

Several studies of comparable cohort size have reported outcomes after use of the omental flap. Shah et al. reported a case series of 11 patients in which an omental flap was used during reoperation for TAGI and reported a 30-day mortality rate of 9.09% and a serious complication rate of 9.09% [9].

Hernandez et al. reported the use of omental flap coverage in 20 patients with TAGI. In that series, overall 30-day mortality was 20%, and 65% of patients experienced a major complication, including anastomotic leak, pseudoaneurysm, omental flap failure/reinfection, or an abdominal complication. In-hospital mortality was 30% [10].

Oda et al. performed a multicenter retrospective review of 68 patients with TAGIs, including 27 patients who underwent omental flap placement. Among the 27 patients who underwent omental flap placement, seven died, for a mortality rate of 25.9%. Despite this high mortality rate, the authors concluded that omental flap coverage was associated with improved outcomes, as mortality was 35.3% for the overall study cohort and 41.4% among patients who did not undergo omental flap placement [11].

Our study demonstrated a 30-day mortality rate of 5.9% and a 30-day major complication rate of 23.5%. This mortality rate was lower than that reported in several previous series using omental flap coverage for complex aortic infections. The current findings should be interpreted cautiously because this was a small, single-center descriptive series without a control group. Nevertheless, the results suggest that a staged approach with mediastinal antibiotic irrigation followed by delayed omental flap coverage is feasible in selected patients.

One notable difference in our method compared with previously reported methods is the inclusion of a mediastinal irrigation catheter for antibiotic infusion after the index operation and before omental flap coverage. Further, our study included only patients who underwent delayed coverage with the omental flap, whereas other studies included both immediate and delayed coverage. In the series by Hernandez et al., delayed coverage was associated with a higher incidence of 30-day mortality (36.4%), and the overall cohort had higher rates of 30-day mortality (20%), major complications (65%), and in-hospital mortality (30%) than observed in our cohort [10]. In contrast, our cohort, which exclusively underwent delayed coverage, had 30-day mortality of 5.9%, a 30-day composite major adverse event rate of 29.4%, and in-hospital mortality of 5.9%.

Management of thoracic aortic infections, recurrent infections, and graft infections has been studied using various strategies, including graft replacement, graft preservation, antimicrobial irrigation, staged reconstruction, and vascularized tissue coverage [12-20]. The available literature remains heterogeneous, with differences in infection definitions, anatomic involvement, operative approach, timing of flap coverage, and outcome reporting. Additional studies with larger cohorts and more standardized definitions are needed to delineate the optimal timing of omental flap coverage and to determine which patients are most likely to benefit from a staged strategy. There are several limitations to this study. The retrospective nature of the study may introduce unaccounted bias. The cohort was small, all patients were male, and there was no control group. In addition, the follow-up period was relatively short, and some patients may develop recurrent infection or require lifetime suppressive antibiotics. The institutional practice described here also limits generalizability, particularly because patient selection and timing of delayed flap coverage were determined by clinical judgment rather than a standardized prospective protocol.

## Conclusions

Recurrent endocarditis and TAGI are rare and complex surgical scenarios, and outcomes have historically been poor for patients with these problems. Various management strategies have been studied, most with high reported mortality rates.

In this small single-center series, mediastinal antibiotic irrigation followed by planned reoperation with mediastinal washout and delayed omental flap coverage was feasible and associated with acceptable short-term mortality and complication rates. Because of the retrospective and descriptive nature of this study, further research with larger cohorts and more standardized controls is needed before this strategy can be broadly recommended as a standard operative protocol.
